# NISQE: Non-Intrusive Speech Quality Evaluator Based on Natural Statistics of Mean Subtracted Contrast Normalized Coefficients of Spectrogram

**DOI:** 10.3390/s23125652

**Published:** 2023-06-16

**Authors:** Shakeel Zafar, Imran Fareed Nizami, Mobeen Ur Rehman, Muhammad Majid, Jihyoung Ryu

**Affiliations:** 1 Department of Computer Engineering, University of Engineering and Technology, Taxila 47050, Pakistan; shakeelzafar@gmail.com (S.Z.); m.majid@uettaxila.edu.pk (M.M.); 2 Department of Electrical Engineering, Bahria University, Islamabad 44000, Pakistan; imran2k2@gmail.com; 3 Department of Electronics and Information Engineering, Jeonbuk National University, Jeonju 54896, Republic of Korea; cmobeenrahman@gmail.com; 4 Electronics and Telecommunications Research Institute (ETRI), Gwangju 61012, Republic of Korea

**Keywords:** speech quality assessment, spectrogram, natural spectrogram statistics, support vector regression

## Abstract

With the evolution in technology, communication based on the voice has gained importance in applications such as online conferencing, online meetings, voice-over internet protocol (VoIP), etc. Limiting factors such as environmental noise, encoding and decoding of the speech signal, and limitations of technology may degrade the quality of the speech signal. Therefore, there is a requirement for continuous quality assessment of the speech signal. Speech quality assessment (SQA) enables the system to automatically tune network parameters to improve speech quality. Furthermore, there are many speech transmitters and receivers that are used for voice processing including mobile devices and high-performance computers that can benefit from SQA. SQA plays a significant role in the evaluation of speech-processing systems. Non-intrusive speech quality assessment (NI-SQA) is a challenging task due to the unavailability of pristine speech signals in real-world scenarios. The success of NI-SQA techniques highly relies on the features used to assess speech quality. Various NI-SQA methods are available that extract features from speech signals in different domains, but they do not take into account the natural structure of the speech signals for assessment of speech quality. This work proposes a method for NI-SQA based on the natural structure of the speech signals that are approximated using the natural spectrogram statistical (NSS) properties derived from the speech signal spectrogram. The pristine version of the speech signal follows a structured natural pattern that is disrupted when distortion is introduced in the speech signal. The deviation of NSS properties between the pristine and distorted speech signals is utilized to predict speech quality. The proposed methodology shows better performance in comparison to state-of-the-art NI-SQA methods on the Centre for Speech Technology Voice Cloning Toolkit corpus (VCTK-Corpus) with a Spearman’s rank-ordered correlation constant (SRC) of 0.902, Pearson correlation constant (PCC) of 0.960, and root mean squared error (RMSE) of 0.206. Conversely, on the NOIZEUS-960 database, the proposed methodology shows an SRC of 0.958, PCC of 0.960, and RMSE of 0.114.

## 1. Introduction

In modern communication systems and mobile networks, speech quality assessment (SQA) has become an integral requirement for system reliability and maintenance of quality of service. With the increase in the transition of voice calls over to internet protocol i.e., voice over internet protocol (VoIP), it is estimated that 85.8% of the internet traffic is due to mobile phones [1]. Communication based on speech requires speech signal acquisition, processing, and transmission. In these steps, a speech signal can be affected by many types of noise. The sources of noise can range from natural environmental factors to limitations in technology or network impairments in the communication systems [2]. SQA can play a vital role in improving the customer’s quality of experience. This can be achieved by the performance evaluation of speech processing systems such as speech coders, automatic speaker recognition systems, speech synthesis systems, etc. Thus, reliable and accurate assessment of speech quality has become a primary requirement of modern multimedia systems for improving customer satisfaction.

SQA can be broadly categorized into two categories: subjective methods [3,4,5,6] and objective methods [7,8,9,10,11,12,13,14,15,16,17]. The most reliable method used for SQA is conducting subjective listening tests utilizing human observers, which normally utilizes mean opinion score (MOS) to measure speech quality, as described in ITU-T P.800 [18]. However, this technique is not suitable for automatic SQA since it is time-consuming and can easily be affected by the user’s prior knowledge of the speech signal and mood of the observer. In contrast, objective SQA methods use a computational model for predicting speech signal quality without involving human observers. Therefore, objective SQA techniques are gaining more importance. Objective SQA is divided into two types: non-intrusive speech quality assessment (NI-SQA) techniques [10,11,19,20,21,22] and intrusive SQA techniques [23,24]. Intrusive SQA algorithms require the pristine clean version of the speech signal as a reference in order to estimate the quality of distorted speech signals [25,26]. Perceptual evaluation of SQA (PESQ) [23] and perceptual objective listening quality assessment (POLQA) [24] are intrusive SQA techniques. The PESQ method predicts the subjective scores of degraded speech samples and returns a score between −0.5 to 4.5, where a higher score indicates higher quality. The PESQ method can also be used specifically for end-to-end network quality assessment. POLQA is an ITU-T standard which is also known as P.863. It is used for SQA in telephone networks and it follows a two-step approach. In the first step, the temporal alignment of the speech signals is performed and the deviation between the pristine and distorted speech signal is computed. In the second step, the quality score of the speech signal is computed.

In a real-time environment, a pristine version of the distorted speech signal is not usually available, so it may be limited in certain applications. On the other hand, NI-SQA techniques do not require a pristine version of the speech signal for the quality estimation of degraded speech signals. This makes the design of NI-SQA techniques more challenging. The standard method for NI-SQA is described in ITU-T Recommendation P.563 [6], but it only shows low correlations with subjective quality ratings when speech quality other than narrow-band transmission is considered [27].

### Related Work

Various NI-SQA techniques are available in the literature. An NI-SQA technique based on neurogram features is proposed in [11]. They use a one-dimensional discrete wavelet transform to compute characteristic frequency responses of each neurogram. The energy of the neurogram is utilized as the input to the support vector regression (SVR) model to perform SQA. In [2], a neural network-based NI-SQA method was proposed that uses the Mel-frequency coefficients with a Gaussian mixture model for the estimation of speech signal quality using a fixed-length matrix of each audio as an input to a convolutional neural network. Mel-frequency features based on cepstral coefficient (MFCC) are extracted [28]. Features based on reconstructed phase space (RPS) are used to estimate the speech signal quality. Mel-frequency coefficients do not perform well in the presence of background noise [29]. In [30], Lyon’s auditory and multi-resolution (MR) features-based non-intrusive model was proposed. The objective MOS is computed using the Gaussian mixture model to estimate the quality of non-intrusive speech signals. The Gaussian mixture model (GMM) uses expectation maximization, which suffers from issues such as false intrinsic mode functions, mode-mixing occurrence, and end effects [31]. Yang et al. used a deep learning approach to NI-SQA that uses the real-time control protocol information to estimate the speech signal quality [32]. Packet loss count, inter-arrival delay, codec delay, delay, jitter, cumulative delay, cumulative jitter, total packet length, and the number of lost packets are used as input features to the deep neural network with six layers. The fixed-size data dimension is obtained by interpolating the input feature vector in the time domain using a deep neural network structure having 32 nodes. Deep learning models have a fixed number of neurons in the input layer. Therefore, the varying size of input data must be reconciled with the fixed size of the input layer. For this purpose, the input data is usually resized, which may cause a loss of important features and information resulting in degradation in the performance of the NI-SQA technique [33].

In [34], a multiresolution auditory model (MRAM) framework for NI-SQA was proposed in which objective MOS of narrowband distorted speech signals are computed utilizing the time-frequency information of the human auditory system. This model uses the multiresolution speech signal with a GMM to extract features. Since this technique also uses GMM, it suffers from false intrinsic mode functions, mode-mixing occurrence, and end effects [31]. Wang et al. proposed an output-based SQA method that uses an autoencoder and SVR to map the feature vector to the objective scores [10]. This technique uses the log power speech spectra to extract features using the autoencoders. The disadvantage of using autoencoders is that they lead to model overfitting [35,36]. In [37], a spectrogram-based convolutional neural network (CNN) for SQA was proposed that makes use of a CNN model to evaluate the speech quality for automatic speech recognition systems. The noise level in a speech signal is determined using the word error and character error rate. Since a CNN requires an input of the same size for each spectrogram, each spectrogram has to be resized to a uniform size. This may lead to the loss of important information and affect the performance of the SQA technique. A natural spectrogram-based non-intrusive SQA model that uses statistical features of the spectrogram to assess the speech quality using SVR was proposed in [19]. Statistical features such as mean, standard deviation, kurtosis, etc., are utilized in the model. Most of the work on SQA focuses on MFCC and MR features. Laboratory-based and crowdsourcing-based SQAs are compared in [38]. Subjective SQA is performed using two methods, i.e., (a) in a laboratory environment and (b) utilizing crowdsourcing. The study concluded that subjective SQA performed using crowdsourcing is less time-consuming as compared to subjective SQA performed in a laboratory environment, but the subjective SQA in a laboratory environment is more reliable. In [39], an SQA technique based on Bayesian non-negative matrix factorization was proposed that utilizes a deep neural network. The technique uses a quasi-clean speech reconstruction to obtain a pseudo-pristine version of the speech signal to perform SQA. The technique is computationally expensive, since the pseudo-reference signal needs to be constructed before SQA can be performed.

ANIQUE+ is an American national standard for non-intrusive estimation of narrowband speech quality. It is based on the perceptual model that utilizes the functional roles of the human auditory system [40]. ANIQUE+ is a complex model that considers articulation analysis, mute detection, and non-speech detection. The classification of speech signals into sub-components (i.e., articulation analysis, mute and non-speech detection) is a complex task, and low classification results may lead to degraded performance. A methodology for SQA in multiple inputs multiple outputs (MIMO) systems was presented in [9]. A speech quality model based on the signal-to-noise ratio, Doppler shift, MIMO configurations, and different modulation schemes is explored. The work emphasized the physical phenomena and explored the impact of an antenna configuration in improving the speech quality for MIMO systems over different modulation schemes.

Most of the aforementioned techniques available in the literature show promising results, but, to the best of our knowledge, none of these techniques use the natural structure of speech signals for assessing speech quality. In this work, a method for NI-SQA is proposed that extracts statistical features from the spectrogram to assess the perceptual speech signal quality. The method is based on natural spectrogram statistics (NSS), which are the statistics extracted from the spectrogram of speech signals. The spectrogram of the pristine speech signal possesses certain statistical properties that hold across different speech contents, and they are disrupted in the presence of distortion or degradation in the speech signal. The presence of distortion in clean speech signals modifies the natural statistical properties of the spectrogram and makes the spectrogram unnatural. The proposed method aims to measure and relate the change in natural statistics of the spectrogram to the perceptual quality of speech signals. The change in statistical properties can be used to assess the perceived speech signal quality. To the best of our knowledge, NSS has not been utilized for predicting the perceived speech signal quality.

The proposed method calculates the natural spectrogram, and the statistical features, i.e., shape, variance, right variance, and mean left variance, in the first step. Then, an SVR is utilized that predicts the perceived quality of speech signals based on the extracted features. An SVR model is used that maps the extracted features to quality scores to produce a non-intrusive SQA. The impact of spectrogram window size on the performance of the proposed methodology is thoroughly investigated. It is demonstrated that the proposed method performs well on independent databases and leads to significant performance improvements when compared with state-of-the-art methods in the literature. The main contributions of this work are twofold:

1. NSS features based on the gradient magnitude and Laplacian of Gaussian are used for SQA;
2. The impact of the spectrogram window size is investigated in detail to assess the optimum window size and percentage of signal overlap.

The remainder of the paper is organized as follows. In Section 2, we present the proposed methodology for NI-SQA. In Section 3, evaluation parameters and performance evaluation of the proposed NI-SQA method are performed. The paper concludes in Section 4, where the conclusion and future work are presented.

## 2. Proposed Methodology for NI-SQA

**Figure 1:**
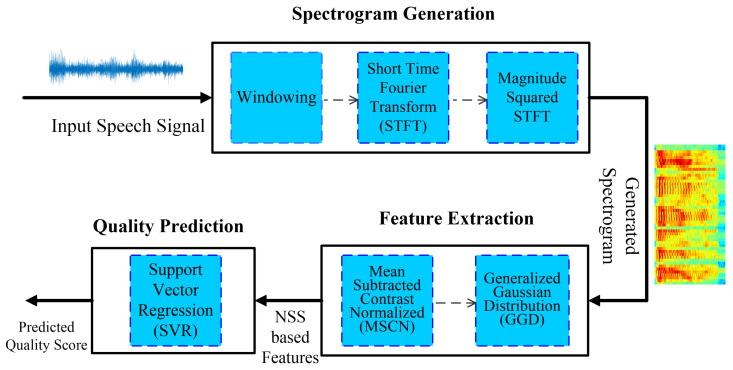
The proposed methodology for NI-SQA based on a speech signal spectrogram.

Figure 1 shows the three-step approach of the proposed methodology for NI-SQA based on NSS features extracted from the spectrogram of speech signals. In the first step, the spectrogram of speech signals is computed using the optimum window size and signal to overlap. In the second step, statistical features from the natural spectrogram statistics using gradient magnitude and Laplacian of Gaussian are extracted. Finally, in the third step, SVR is used for predicting the speech quality score. Each step of the proposed NI-SQA is described in detail below.

### 2.1. Spectrogram Generation

**Figure 2:**
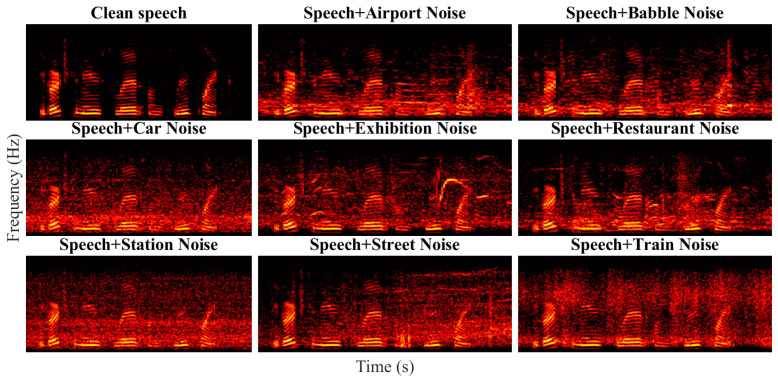
Spectrogram plot comparison of clean speech and distorted speech signal over different types of noises.

Speech signals can be represented visually using a spectrogram, which is computed using the Fourier transform over a short time window. A speech spectrogram has been a fundamental instrument for gaining an understanding of how the sounds of speech are produced. A spectrogram of pristine speech signals maintains certain statistical properties (shape, mean, and variance), which are altered when distortion is present in the speech signal. Figure 2 shows the spectrogram generated from a clean speech signal and its noisy version from eight different noises, i.e., babble, airport, exhibition, car, station, restaurant, train, and street. It can be observed that the spectrograms are visually different and show a unique pattern depending on the noise type. When the deviations between spectrogram statistics of the pristine and distorted speech signal are quantified appropriately, these statistics can be utilized to assess the quality of the speech signal without using the pristine version of the speech signal.

The spectrogram of any finite duration discrete-time signal f[a] can be obtained using discrete Fourier transform (DFT), which is given as follows:

(1) $$\hat{Y}\left[l\right]=\sum_{a=0}^{N-1}f\left[a\right]e^{-i\frac{2\pi l}{N}a},\;l=0,\ldots ,N-1,$$

where $\hat{Y}\left[l\right]$ is the DFT of f[a] and *N* is the total number of samples. The inverse DFT is given as shown:

(2) $$f\left[a\right]=\frac{1}{N}\sum_{l=0}^{N-1}\hat{Y}\left[l\right]e^{-i\frac{2\pi a}{N}l},\;a=0,\ldots ,N-1.$$

We can represent these equations in the form of matrices:

(3) $$\begin{array}{c} f=\frac{1}{N}M\hat{Y},\\ \hat{Y}=\bar{M}f, \end{array}$$

where $\bar{M}$ represents the complex conjugate of *M*, and *M* is the N×N Fourier matrix:

$$M=\left(\begin{array}{ccccc} 1 & 1 & 1 & \cdots & 1\\ 1 & e^{i\frac{2\pi }{N}} & e^{i\frac{4\pi }{N}} & \cdots & e^{i2\pi \frac{N-1}{N}}\\ \vdots & \vdots & \vdots & \ddots & \vdots \\ 1 & e^{i2\pi \frac{N-1}{N}} & e^{i2\pi \frac{2(N-1)}{N}} & \cdots & e^{i2\pi \frac{{\left(N-1\right)}^{2}}{N}} \end{array}\right)$$

This DFT can be represented graphically using the magnitude of $\hat{y}$ for frequencies on the range $\left[-F_{s}/2,F_{s}/2\right]$. In our case, let *f* be a speech signal having length *N*. When considering the consecutive segments of speech signal *f*, i.e., ${\left[f\left[0\right],f\left[1\right],\cdots ,f\left[m-1\right]\right]}^{T}$ represents the first column in *f*, ${\left[f\left[1\right],f\left[2\right],\cdots ,f\left[m\right]\right]}^{T}$ represents the second column in *f*, and so on. It is observed that the index of both columns and rows in *Y* is time. It can also be observed that *Y* represents *f* in a highly redundant manner. The spectrogram with window size *m* is given as shown:

(4) $$\begin{array}{c} \hat{Y}=\bar{M}f,\\ Y=\frac{1}{m}M\hat{Y}. \end{array}$$

The columns of $\hat{Y}$ are indexed by time and rows are indexed by frequency.

### 2.2. Feature Extraction

The proposed NI-SQA method is based on the generalized Gaussian distribution (GGD). It is based on the assumption that the mean subtracted contrast normalized (MSCN) spectrogram coefficients of the natural spectrogram have certain statistical properties that are changed in the presence of noise. By quantifying these statistical changes in distorted spectrograms, we can predict the quality of distorted speech signals. Such techniques have been applied successfully in blind image quality assessment methods [41]. In the first step, locally normalized Fourier transform via divisive normalization and local mean subtraction are computed from degraded speech spectrograms. To normalize the local variance and reduce the autocorrelation within a signal, Ruderman [42] observed that a local non-linear operation to log contract Fourier transform to remove local mean displacement can be utilized. Such an operation can easily be applied to a given distorted spectrogram. The MSCN coefficients of the distorted spectrogram are computed as follows:

(5) $$MC\left(m,\Omega \right)=\frac{\hat{Y}\left(m,\Omega \right)-\mu \left(m,\Omega \right)}{\delta \left(m,\Omega \right)+k_{1}},$$

where *m* is the window size, $\hat{Y}\left(m,\Omega \right)$ is the input spectrogram, μ(m,Ω) represents the mean, δ(m,Ω) represents the standard deviation of the spectrogram, and $k_{1}$ is a constant, given as follows:

(6) $$\mu =\sum_{k=-E_{1}}^{E_{1}}\sum_{l=-L}^{L}\Omega_{k,l}I_{k,l}\left(m,\Omega \right),$$

where $\Omega_{k,l}$ are positive weights and $I_{k,l}$ are the spectrogram coefficients.

(7) $$\sigma \left(m,\Omega \right)=\sqrt{\sum_{k=-E_{1}}^{E_{1}}\sum_{l=-L}^{L}A_{k,l}{\left(I_{k,l}\left(m,\Omega \right)-\mu \left(m,\Omega \right)\right)}^{2}},$$

where *A* is a two-dimensional circularly-symmetric Gaussian weighting function that is sampled at three standard deviations and then re-scaled to a unit volume, $E_{1}$ and *L* are taken as 3, and σ and μ are the standard deviation and mean of the window of size (E−1)×L.

**Figure 3:**
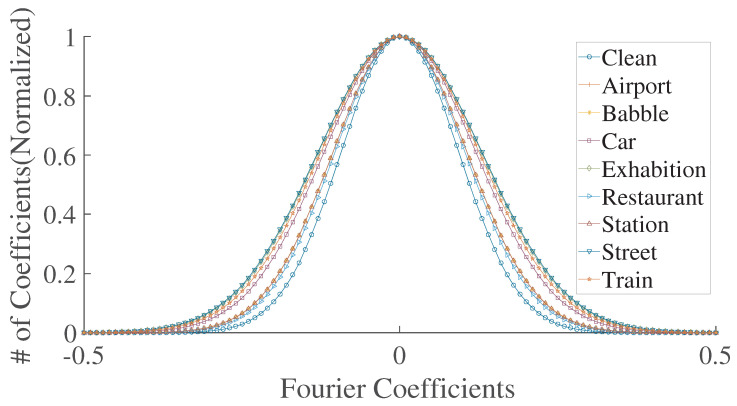
Gaussian curve statistics from standard deviation of the clean and distorted spectrograms in Figure 1 for different distortions.

To determine that statistics are affected by each distortion in a particular fashion, Figure 3 plots the coefficient distributions from the standard deviation of the distorted spectrogram feature vector for each distortion considered in this work. It can be observed that each distortion affects the statistics of the spectrogram characteristically. As MSCN coefficients are symmetric, a zero-mean Gaussian distribution is used, which is given as follows:

(8) $$G\left(i;\beta ,\sigma^{2}\right)=\frac{\beta }{2\alpha \gamma (\frac{1}{\beta })}exp\left(-{\left(\frac{\left|i\right|}{\alpha }\right)}^{\beta }\right),$$

where $\sigma^{2}$ controls the variance and β controls the shape of GGD. We have used the moment matching-based approach for the estimation of GGD parameters, where α and γ are given as follows:

(9) $$\alpha =\sigma \sqrt{\frac{\gamma (1/\beta )}{\gamma (3/\beta )}},$$

and the gamma function γ(·) is given by the following:

(10) $$\gamma \left(x\right)=\int_{0}^{\infty }t^{x-1}e^{t}dt,\;a>0.$$

The MSCN coefficients are symmetric; therefore, zero-mean Gaussian distribution was selected. The parameters γ and σ are computed using a moment-matching-based approach for each signal.

The estimated parameters of GGD are used to compute the MSCN distributions from distorted and pristine speech signals. For each speech signal, two parameters from the GGD fit and the statistical relationship between two neighboring normalized Fourier transform coefficients are computed. The relationship between the pairwise products of neighboring MSCN coefficients along with four directions, i.e., vertical ($V_{c}$), horizontal ($H_{r}$), secondary diagonal ($D_{s}$), and main diagonal ($D_{m}$), are computed as follows:

(11) $$\begin{array}{c} H_{r}\left(r,s\right)=\tilde{\hat{Y}}\left(r,s\right)\tilde{\hat{Y}}\left(r,s+1\right),\\ V_{c}\left(r,s\right)=\tilde{\hat{Y}}\left(r,s\right)\tilde{\hat{Y}}\left(r+1,s\right),\\ D_{m}\left(r,s\right)=\tilde{\hat{Y}}\left(r,s\right)\tilde{\hat{Y}}\left(r+1,s+1\right),\\ D_{s}\left(r,s\right)=\tilde{\hat{Y}}\left(r,s\right)\tilde{\hat{Y}}\left(r+1,s-1\right), \end{array}$$

where the neighboring statistical relationships along with four orientations are represented by vertical ($V_{c}$), horizontal ($H_{r}$), secondary diagonal ($D_{s}$), and main-diagonal ($D_{m}$), and u∈1,2,…,M1 and v∈1,2,…,N1 are spatial indices. The NSS features of the speech spectrogram are extracted at two scales.

### 2.3. Quality Prediction

In the third step, the extracted features are used to assess the speech quality score. The extracted features of speech signals are given as inputs to the SVR algorithm to predict the speech quality score. The SVR model is given as follows:

(12) $$\psi \left(S_{D}\right)=\alpha_{1}\beta_{1}\left(S_{D}\right)+c,$$

where $S_{D}$ is the extracted feature in vector form, $\beta_{1}$ represent the feature space, and $\alpha_{1}$ and *c* represent the weight constant and bias value, respectively. LibSVM package was used to implement the SVR model [43].

## 3. Experimental Results

### 3.1. Database Description and Training Setup

In this work, two databases, namely, the Center for Speech Technology voice cloning toolkit Corpus (VCTK-Corpus) [44] and NOIZEUS-960 database [45], were used. The VCTK-Corpus includes the speech data of different accents pronounced by 109 English speakers. The selected speech signals were taken from sentences from the newspaper and the rainbow passage. All speech signals were recorded using the same recording setup—96 kHz sampling frequency at 24 bits—at the University of Edinburgh. All speech signals were converted into 16 bits and downsampled to 48kHz. VCTK-Corpus consists of a total of 44,242 speech utterances. In our experiments, VCTK-Corpus downsampled at 8 kHz was used. We took a subset of 3270 clean speech signals from all the passages, which were then corrupted by 8 different types of noise, i.e., babble, airport, exhibition, car, train station, restaurant, train, and street, at 4 different noise levels, i.e., 15 dB, 10 dB, 5 dB, and 0 dB. A total of 104,640 degraded speech signal samples were used for experiments. The NOIZEUS-960 database consists of 30 clean speech signals, which were corrupted by the same 8 different types of noise locations, i.e., babble, airport, exhibition, car, train station, restaurant, train, and street, and at 4 distinct distortion/noise levels, i.e., 15 dB, 10 dB, 5 dB, and 0 dB.

To verify the quality assessment results and ensure reliable comparison, the dataset was divided into two subsets, i.e., 80% were used for training and 20% were used for testing. The samples for both training and testing in the SVR model were disjointed, such that the samples used in the training were not present in the testing subset. The training and testing were performed over 1000 iterations to remove the performance bias due to the random selection of testing and training samples, and the median scores over all the iterations are reported.

The quality evaluation of the speech signals was performed using perceptual evaluation of speech quality (PESQ), which is an ITU standard (P.862) for end-to-end speech quality assessment [46]. The PESQ is a full reference SQA model, and it can predict the subjective quality of speech signal with good correlation across various conditions. The MOS scores obtained utilizing PESQ are termed as PESQ–MOS in this work.

### 3.2. Performance Evaluation Criteria

Three parameters were used for the evaluation of the proposed methodology, i.e., Spearman’s rank-ordered correlation coefficient (SRC), Pearson correlation coefficient (PCC), and root mean squared error (RMSE). SRC is represented as follows:

(13) $$SRC=\frac{6\sum_{j=1}^{Z}S_{i}}{Z(Z^{2}-1)},$$

where $S_{i}$ is the difference between the ranks of *j*th speech signal subjective and predicted quality score ranks, and *Z* is the total number of samples. A value of SRC close to unity, i.e., 1, represents a higher correlation between predicted and subjective quality scores, whereas an SRC value close to zero represents a low correlation. The second parameter used to evaluate the proposed methodology for NI-SQA is the Pearson correlation coefficient (PCC) which is represented below [47]:

(14) $$PCC=\frac{\sum_{j=1}^{Z}\left(t_{i}-\bar{t}\right)\left(r_{i}-\bar{r}\right)}{\sqrt{\sum_{j=1}^{Z}{\left(t_{i}-\bar{t}\right)}^{2}{\left(r_{i}-\bar{r}\right)}^{2}}},$$

where $r_{i}$ represents the subjective quality score and $t_{i}$ represents the quality score predicted by the proposed methodology. A PCC score value close to unity, i.e., 1, represents a higher correlation between the predicted and subjective quality score. The third parameter used to evaluate the proposed methodology is the root mean squared error (RMSE) between the objective and subjective quality scores. An RMSE value close to zero represents a higher correlation between the perceptual quality and predicted MOS of the speech signal. RMSE is represented as shown:

(15) $$RMSE=\sqrt{\frac{\sum_{j=1}^{Z}{\left(L_{i}-\hat{L_{i}}\right)}^{2}}{Z}},$$

where $L_{i}$ represents the subjective MOS and $\hat{L_{i}}$ represents the quality score predicted using the proposed methodology.

### 3.3. Performance Analysis

**Figure 4:**
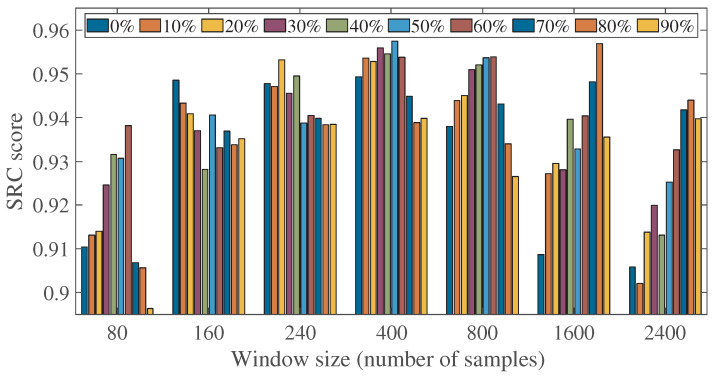
Comparison of SRC score with different window length and overlapping samples in windows.

**Figure 5:**
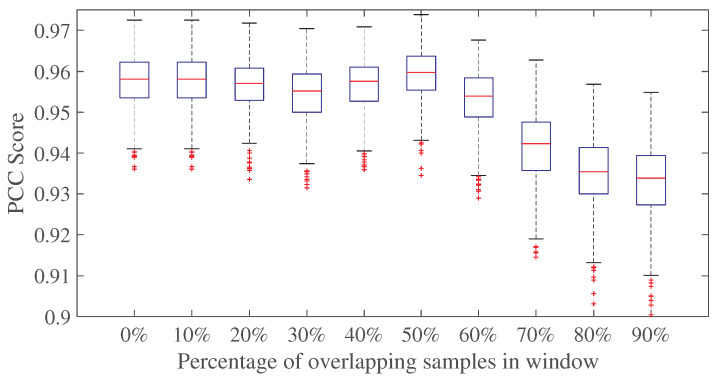
Box plot of PCC scores using optimum length of window 400 with 0 to 90 percent overlapping samples in windows.

Analysis was performed to determine the optimum window size and signal to overlap for the proposed NI-SQA. Different window sizes and percentages of overlapping samples concerning speech spectrograms were extracted. Figure 4 shows the SRC scores obtained using the proposed NI-SQA methodology at different window sizes, ranging between 80 and 2400 samples, and different percentages of signal, overlapping between 0% and 90%. It can be observed from Figure 4 that the window length and overlapping in samples have an impact on the SRC scores of the proposed method. It can also be observed that the optimal parameters for the spectrogram computation are a window size of 400 samples with 50% overlapping between the windows. Figure 5 shows the box plot in terms of PCC scores over 1000 iterations of the proposed methodology, with a window size of 400 samples and sample overlapping ranging between 0% and 90%. It can be observed that the box plot with the highest median PCC score is obtained with an overlap of 50%.

**Figure 6:**
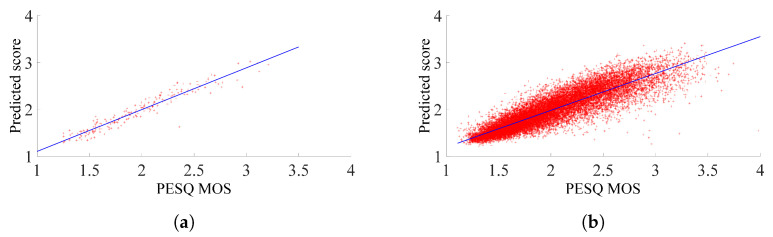
Scatter plot of the proposed NI-SQA applied to (**a**) NOIZEUS-960 database, (**b**) VCTK-Corpus database.

Figure 6a,b shows scatter plots of quality scores obtained with the proposed method vs. corresponding PESQ–MOS values. The diagonal line represents the highest correlation between the PESQ–MOS and predicted MOS, where the predicted and subjective quality scores are equal. It can be observed from Figure 6a,b that the predicted score aligns closely with the diagonal line, which means that there is a high correlation between predicted and PESQ–MOS scores.

Table 1 shows the performance comparison of the proposed methodology on individual noise type over the NOIZEUS-960 database in terms of PCC score with four state-of-the-art SQA techniques. The bold values represent the top-performing technique. The results indicate that the performance of the proposed method is ranked as the top for all the distortion types except for train noise, where the PCC score of MRAM+ [48] is ranked top, i.e., 0.894, and the proposed methodology is ranked third with a PCC score of 0.842. The proposed NI-SQA was compared with ITU-T P.563, Lyon’s model, MRAM features model, and LSF-based [10] model. It can be observed that, for the airport, exhibition, restaurant, and street distortion types, the proposed methodology is ranked as the top, with PCC scores of 0.957, 0.923, 0.932, and 0.920, respectively, and MRAM+ [48] is ranked second, with PCC scores of 0.892, 0.855, 0.894, and 0.855, respectively. For the babble and station distortion types, the proposed methodology is again ranked at the top, with PCC scores of 0.941 and 0.921, and MRAM+ [48], MRAM, MFCC+LSF [10] are ranked second with PCC scores of 0.924 and 0.864, respectively. For the car noise type, the proposed methodology is ranked at the top, whereas MRAM, MFCC+LSF [10] is ranked second, with PCC scores of 0.95 and 0.909, respectively.

Table 2 shows the overall performance comparison of the proposed methodology with nine state-of-the-art SQA techniques. The bold values represent the top-performing technique. It can be observed that the proposed technique shows the best performance over the NOIZEOUS-960 database with an SRC score of 0.958, a PCC score of 0.960, and an RMSE of 0.114. i-vector VQ model [49] is ranked second, with a PCC score of 0.950 and an RMSE of 0.210. The proposed methodology is ranked first in the VCTK-Corpus database, with an SRC score of 0.902, a PCC score of 0.891, and an RMSE of 0.206. NSS SQA [19] is ranked second, with an SRC score of 0.894, a PCC score of 0.894, and an RMSE of 0.213.

### 3.4. Discussion

In Table 1, the performance of the proposed method is ranked at the top for all the distortion types except for train noise, where the PCC score of MRAM+ [48] is ranked at the top and the proposed methodology is ranked third. The reason behind this discrepancy can be attributed to the inherent complexity and uniqueness of the train noise patterns present in the dataset. Our proposed method, although effective in handling various types of noise, might not have been optimized specifically for the train noise characteristics, as the train noise follows periodic patterns and the spectrograms generated from train noise have less intra-class variation. As a result, its performance in mitigating the train noise may not have been as strong as that of existing methods. Therefore, the proposed methodology performs better on those types of noise. In Table 2, the proposed method performs well in comparison to i-vector framework [16] on the NOIZEOUS-960 database, which is a full reference SQA technique.

## 4. Summary, Conclusions, and Future Work

NI-SQA has gained importance due to the rise in the usage of speech-processing algorithms in multimedia applications. NI-SQA is a challenging task, due to advancements in technology, an increase in the use of multimedia content in daily life, and the absence of reference speech. This work proposes a novel NI-SQA method that utilizes the local NSS features and predicts the quality score using SVR. The NSS of pristine speech signals has certain characteristics that are disrupted in the presence of distortion. The experimental results show that the NSS is useful for performing SQA. The selection of optimal window size and signal overlap helped in improving the performance of the proposed methodology. It can be seen that the proposed method shows better performance in comparison with state-of-the-art NI-SQA techniques, which is evident from the results. The experimental results show that the proposed methodology utilizes the natural structure of speech signals effectively for SQA. The NSS obtained from the speech spectrogram can be used for assessing speech quality. In future work, CNN can be utilized for SQA on the extracted spectrograms. Furthermore, self-supervised learning models in artificial intelligence can be utilized to support SQA. Future work can include diverse kinds of distortions due to environmental factors in the transmission channel, noise introduced due to low bit rate speed codecs, and noise induced due to different modulation techniques.

## Figures and Tables

**Table 1:** Performance comparison on individual distortion types, in terms of PCC, of the proposed NI-SQA methodology with state-of-the-art approaches.

Database	Types of Distortions	ITU-T Rec. P.563 [6]	Lyon + MRAM [30]	MRAM + Features [48]	MRAM, MFCC and LSF [10]	Proposed Model
NOIZEUS-960	Airport	0.694	0.770	0.892	0.874	**0.957**
	Babble	0.790	0.829	0.924	0.924	**0.941**
	Car	0.788	0.819	0.890	0.909	**0.950**
	Exhibition	0.725	0.705	0.855	0.847	**0.923**
	Restaurant	0.622	0.798	0.894	0.885	**0.932**
	Station	0.597	0.745	0.864	0.864	**0.921**
	Street	0.736	0.751	0.855	0.830	**0.920**
	Train	0.813	0.808	**0.894**	0.873	0.842

**Table 2:** Performance comparison of the proposed NI-SQA methodology with state-of-the-art approaches in terms of SRC, PCC, and RMSE.

Technique	Database	SRC	PCC	RMSE
ITU-T Rec. P.563 [6]	NOIZEUS-960	-	0.717	
Lyon + MRAM [30]	NOIZEUS-960	-	0.883	0.326
i-Vector Framework [16]	NOIZEUS-960	0.900	-	0.300
MRAM + MFCC [34]	NOIZEUS-960	0.854	-	0.368
NSQM [11]	NOIZEUS-960	-	0.880	0.210
i-vector average model [49]	NOIZEUS-960	-	0.890	0.240
i-vector VQ model [49]	NOIZEUS-960	-	0.950	0.210
ANIQUE+ [40]	NOIZEOUS-960	0.886	0.890	0.324
ANIQUE+ [40]	VCTK-Corpus	0.842	0.849	0.301
NSS SQA [19]	NOIZEUS-960	0.920	0.922	0.159
NSS SQA [19]	VCTK-Corpus	0.894	**0.894**	0.213
Proposed	NOIZEUS-960	**0.958**	**0.960**	**0.114**
Proposed	VCTK-Corpus	**0.902**	0.891	**0.206**
